# Hypotonicity differentially affects inflammatory marker production by nucleus pulposus tissue in simulated disc degeneration versus herniation

**DOI:** 10.1002/jor.24268

**Published:** 2019-04-01

**Authors:** Vivian H. M. Mouser, Irene T. M. Arkesteijn, Bart G. M. van Dijk, Karin Wuertz‐Kozak, Keita Ito

**Affiliations:** ^1^ Orthopaedic Biomechanics Department of Biomedical Engineering Eindhoven University of Technology Eindhoven The Netherlands; ^2^ Institute for Biomechanics Department of Health Sciences and Technology ETH Zurich Zurich Switzerland; ^3^ Department of Health Sciences University of Potsdam Potsdam Germany; ^4^ Schön Clinic Munich Harlaching (Academic Teaching Hospital and Spine Research Institute of the Paracelsus Medical University in Salzburg) Munich Germany; ^5^ Department of Orthopedics University Medical Center Utrecht Utrecht The Netherlands

**Keywords:** nucleus pulposus, inflammation, intervertebral disc degeneration, (hypo)tonicity

## Abstract

Inflammatory cytokines play an important role in intervertebral disc degeneration. Although largely produced by immune cells, nucleus pulposus (NP) cells can also secrete them under various conditions, for example, under free swelling. Thus, tissue hypotonicity may be an inflammatory trigger for NP cells. The aim of this study was to investigate whether decreased tonicity under restricted swelling conditions (as occurring in early disc degeneration) could initiate an inflammatory cascade that mediates further degeneration. Healthy bovine NP tissue was balanced against different PEG concentrations (0–30%) to obtain various tissue tonicities. Samples were then placed in an artificial annulus (fixed volume) and were cultured for 3, 7, or 21 days, with free swelling NP as control. Tissue content (water, glycosaminoglycan, collagen) was analyzed, and both the tissue and medium were screened for tumor necrosis factor alpha (TNF‐α), interleukin‐1β (IL‐1β), interleukin‐6 (IL‐6), interleukin‐8 (IL‐8), prostaglandin‐E_2_ (PGE_2_), and nitric oxide (NO). A range of tonicities (isotonic to hypotonic) was present at day 3 in the PEG‐treated samples. However, during culture, the tonicity range narrowed as GAGs leached from the tissue. TNF‐α and IL‐1β were below detection limits in all conditions, while mid‐ and downstream inflammatory cytokines were detected. This may suggest that the extracellular environment directly affects NP cells instead of inducing a classical inflammatory cascade. Furthermore, IL‐8 increased in swelling restricted samples, while IL‐6 and PGE_2_ were elevated in free swelling controls. These findings may suggest the involvement of different mechanisms in disc degeneration with intact AF compared to herniation, and encourage further investigation. © 2019 The Authors. *Journal of Orthopaedic Research*® Published by Wiley Periodicals, Inc. on behalf of Orthopaedic Research Society. J Orthop Res

Low back pain is the most common cause of chronic pain in the aging western world.^1^, ^2^ The total number of years lived with disability due to low back pain is still increasing.^2^ Moreover, the incidence of low back pain shows a strong correlation with intervertebral disc degeneration.^3^ However, current therapies to treat pain in an early stage of disc degeneration only have a 50% success rate on the long‐term.^4^ To improve early intervention strategies, a better understanding of the development and progression of disc degeneration is essential.

The healthy intervertebral disc consists of a gel‐like core, the nucleus pulposus (NP), which is circumferentially surrounded by several highly organized fibrous layers which together form the annulus fibrosus (AF). The NP and inner AF are cranially and caudally separated from the vertebrae by the porous hyaline cartilage endplates, through which nutrients diffuse. Several factors are of influence for disc degeneration, for example, genetic inheritance, impaired nutrient supply, and mechanical loading. However, the exact cause and developmental path are still unclear.^5^, ^6^, ^7^ During disc degeneration, the metabolism of the NP cells is altered, resulting in a decrease in proteoglycan and collagen type II synthesis, and an increase in collagen type I synthesis, metalloproteinase (MMP) activity, and/or a disintegrin and metalloproteinase with thrombospondin motifs (ADAMTS) activity.^6^ Consequently, the fixed charge density generated by the negatively charged chondroitin and keratan sulfate groups of the proteoglycans is reduced. This reduction impairs the overall swelling pressure and thus the load‐bearing capacity of the NP, resulting in a loss of disc height and overloading of the AF. With this overloading, the AF becomes damaged and disorganized, and in advanced disc degeneration, macroscopic tears are frequent.^5^, ^8^, ^9^ As tears coalesce and grow, the AF may rupture, and the NP will extrude out of the intervertebral disc (herniation), causing severe pain for the patient due to the compression and irritation of nerves and inflammation of the surrounding tissue.

Although inflammation is most profound after herniation, pro‐inflammatory cytokines, such as tumor necrosis factor alpha (TNF‐α), interleukin‐1β (IL‐1β), interleukin‐6 (IL‐6), and prostaglandin‐E_2_ (PGE_2_), are also detected in NP tissue without herniation,^10^, ^11^, ^12^, ^13^, ^14^ suggesting a crucial role of inflammation in the process of disc degeneration.^6^, ^15^ Especially TNF‐α and cytokines of the interleukin family have been associated with disc degeneration and are known to trigger an inflammatory cascade, which downstream causes an upregulation of genes that encode matrix‐degrading enzymes.^15^ Moreover, the presence of inflammatory factors seems to be related to pain in an early stage of disc degeneration when the AF is still intact, the so‐called discogenic pain.^6^

The pro‐inflammatory cytokines, present in extruded NP tissue, are mainly attributed to the body's immune response.^16^, ^17^, ^18^, ^19^, ^20^ However, NP cells themselves are also capable of producing inflammatory cytokines in vitro^21^, ^22^, ^23^ and in vivo.^16^, ^24^ In addition, previous work demonstrated a sustained inflammatory response when NP tissue was cultured in vitro under unconstrained, free swelling (FS) conditions that serve as a model for herniation.^25^ These findings suggest that a hypotonic environment, due to free swelling, triggers an inflammatory response in the NP cells. Possibly, a decrease in tonicity due to the loss of proteoglycans during early disc degeneration will also trigger an inflammatory response of the NP cells. The identification of inflammation triggers for the NP could provide insights into the mechanism of disc degeneration progression and could be used to improve treatment modalities for early disc degeneration. Therefore, the aim of this study was to evaluate whether a decrease in tonicity of constrained NP tissue initiates an inflammatory cascade which mediates tissue degeneration. To evaluate this hypothesis, NP tissue was cultured under various tonicities using an ex vivo constrained NP model, and both the tissue and its released products were analyzed.

## MATERIALS AND METHODS

### Tissue Culture

**Figure 1:**
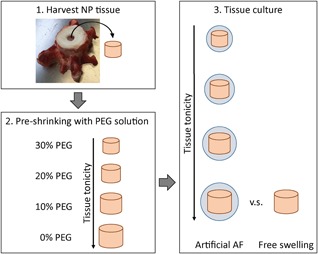
Schematic overview of the experimental setup. First, NP tissue was harvested from bovine tails. Following, tissues were placed in dialysis tubing to be shrunken by balancing the tissue for 100 min to PBS with 0, 10, 20, or 30% PEG. After shrinking, samples contained various tonicities and were placed in an artificial AF to be cultured for 21 days. As a control, samples were cultured without being placed in the artificial AF to allow free swelling.

Bovine caudal discs (cd1‐cd5; 24‐months old) were obtained from the local abattoir in accordance with local regulations. The discs were transversally opened at the cartilage endplate/NP interface, and NPs were harvested from the central part of the disc with a biopsy punch (8 mm diameter, Kruuse, Sherburn, UK). From each tail, one NP was weighed and snap frozen directly after harvesting as base‐line measurement (day 0). The other NPs were weighed and equally distributed (disc level per donor) over the culture groups (Table S1). Next, each NP was placed in dialysis tubing (15 kDa molecular weight cut‐off, Spectra‐Por, Rancho Dominguez, CA) and pre‐shrunk in 30%, 20%, 10%, or 0% w/v polyethylene glycol (PEG, 20 kDa) in PBS for 100 min, depending on the assigned group (Tables S1 and S2). Thereafter, the dialysis tubing was removed, NPs were weighed, wrapped tightly in a 0.2 µm pore size regenerated cellulose membrane (Whatman, Sigma, Zwijndrecht, The Netherlands), and placed in an artificial annulus consisting of custom jackets (Varodem, Saint‐Leger, Belgium) knitted from Dyneema Purity fibers (DSM, Heerlen, the Netherlands).^26^ The artificial annulus was closed with needle and thread. This system has been demonstrated to maintain healthy NP tissue in long‐term culture when pre‐shrinking the tissue to generate a physiological tonicity.^26^ An unconstrained (free swelling, FS) group was used as a control^25^ (Fig. 1). Samples were cultured in deep, non‐coated 12‐well plates (PS‐Thinsert Plate, Greiner Bio‐One Gmbh, Alphen a/d Rijn, the Netherlands) with 6 ml of culture medium consisting of Glutamax Dulbecco's modified Eagle medium (DMEM, 21885, Gibco, Invitrogen, Bleiswijk, the Netherlands), 3% fetal bovine serum (FBS, 758073, Greiner Bio‐One), 50 μg/ml ascorbic acid (l‐ascorbic acid 2‐phosphate, A8960, Sigma), and 1% penicillin/streptomycin (DE17‐602E, Lonza, Basel, Switzerland) under physiological conditions (37°C, 5% O_2_, and 10% CO_2_, i.e., pH = 7.2). Medium was changed twice a week, collected, and stored at −80°C until further use. At days 3, 7, and 21, NP samples were removed from the artificial annulus, weighed, snap frozen, and stored at −80°C until further use.

### Biochemical Content in Tissue and Medium

To assess the biochemical content in the tissue, one‐fourth of each NP sample was weighed (wet weight), lyophilized (Freezone 2.4, Labconco, Kansas City, MO) overnight and weighed again (dry weight). The water content was calculated by dividing the difference between wet and dry weight by the wet weight. Subsequently, the samples were digested overnight in papain digestion buffer (100 mM phosphate buffer, 5 mM l‐cystein, 5 mM ethylenediaminetetra‐acetic acid, and 140 µg/ml papain, all from Sigma) at 60°C. The DNA content of the digested samples was measured with the Qubit Fluorometric Quantitation method (Thermo Fisher Scientific, Landsmeer, the Netherlands). The glycosaminoglycan (GAG) content, as a measure for the amount of proteoglycans, was determined with a DMMB assay^27^ using known concentrations of shark cartilage chondroitin sulfate (C4384, Sigma) as reference. The tonicity was calculated from the fixed charge density, which is a measure of the GAG per wet weight, as described by Narmoneva et al.^28^ and Huyghe et al.^29^ The hydroxyproline (HYP) content was measured with a chloramin‐T assay^30^ using trans‐4‐hydroxyproline (H5534, Sigma) as reference. To assess the proteoglycan content in the medium, medium samples were digested overnight in 50% v/v papain digestion buffer at 60°C. The GAG content was determined with the DMMB assay^27^ with a standard curve made of 50% v/v culture medium.

### Cytokine Concentrations in Tissue and Medium

To assess the cytokine concentration in the tissue, half of each tissue sample was pulverized with a mikro‐dismembrator (Sartorius, Goettingen, Germany), as described by van Dijk et al.^26^ To each sample 1 ml of EDTA‐free cOmplete Lysis‐M buffer (Roche, Almere, the Netherlands) was added, and samples were incubated overnight at 4°C and stored at −80°C. The cytokine concentrations in the tissue were measured with bovine specific ELISA kits for TNF‐α (detection limit: 125.0 pg/ml, R&D Systems, Abingdon, UK), IL‐1β (detection limit: 31.3 pg/ml, Thermo Fisher Scientific and MyBioSource, Uithoorn, the Netherlands), IL‐6 (detection limit: 78.1 pg/ml, Thermo Fisher Scientific), and IL‐8 detection limit: 8 pg/ml, Mabtech, Nacka Strand, Sweden).

Furthermore, the concentrations of TNF‐α, IL‐1β, IL‐6, IL‐8, and PGE_2_ (detection limit: 13.4 pg/ml, Enzo Life Sciences, Raamdonksveer, the Netherlands) were measured in the medium by ELISA. The concentrations were normalized to the initial sample wet weights to obtain the overall tissue response. If possible, standard curves were prepared in culture medium to account for medium effects. If this was not possible, the cytokine concentration in the medium was determined as control on the plate.

### Nitric Oxide Concentrations in Medium

The nitric oxide (NO) concentration in the medium (N = Table S1) was measured as described by Miranda et al.^31^ Briefly, vanadium chloride (Sigma) and Griess reagent (Sigma) were added to the medium, followed by incubation at 37°C for 30 min. Nitrite and nitrate (both Sigma) standards were read on the same plate.

### Statistics

Statistical analysis was performed using R‐project software (version 3.0.2).^32^ Due to unbalanced and low number of independent samples per group and time point (Table S1), non‐parametric statistical analysis was performed. Although the experimental design is two‐way (tonicity condition and time), no two‐way non‐parametric analysis was available. Hence, Kruskal–Wallis tests and Mann–Whitney U post‐hoc testing with Bonferonni correction were used to analyze the differences between groups (each time point) or the time‐dependent effects within a group (each group). Statistical significance was assumed for *p* < 0.05.

## RESULTS

### Tissue Tonicity and Water Content

**Figure 2:**
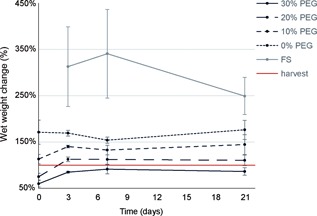
Wet weight change normalized to the initial weight, for samples balance to the different PEG concentrations (day 0) and during culture. 30%, 20%, 10%, and 0% refer to the [PEG] used to pre‐shrink the NP samples, before constraint in the artificial annulus. FS = free swelling, unconstrained NP tissue. Red line indicates the sample wet weight directly after harvest before pre‐shrinking (100%).

Using the PEG system for pre‐shrinking of the samples, the wet weight of the samples could be initially (day 0) adjusted to a range from 59 ± 7% to 171 ± 26% of the initial wet weight (Fig. 2). During the first 3 days of culture, the wet weight of the samples balanced against 10–30% PEG slightly increased (132 ± 10% to 140 ± 2%; 75 ± 7% to 112 ± 5%; 59 ± 7% to 84 ± 2%, for the 10%, 20%, and 30% PEG groups, respectively), whereas the wet weight of the free swelling samples increased three‐fold during this period.

**Figure 3:**
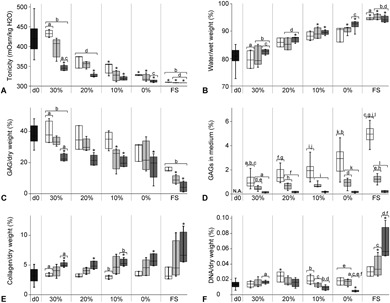
Water and biochemical content of cultured NP tissues. (A) Tissue tonicity, (B) water content per wet weight, (C) proteoglycan content per dry weight, (D) proteoglycans in the medium per 3 days, normalized to the initial tissue weight, (E) collagen content per dry weight, and (F) DNA content per dry weight. X‐axis: d0 = native control, values indicated with black bars. 30%, 20%, 10%, and 0% refer to the [PEG] used to pre‐shrink the NP samples, before constraint in the artificial annulus. FS = free swelling, unconstrained NP tissue. White bars = day 3, light gray bars = day 7, and dark grey bars = day 21. See Table S1 for n per group. * = significantly different (*p* < 0.05) from day 0. a,b,c,.. = significantly different (*p* < 0.05) from bars with the same letter.

The tonicity of the samples balanced to 0% and 10% PEG, was lower on days 3 and 7 than on day 0. This was not the case for the 20% and 30% PEG groups (Fig. 3A). Furthermore, the tonicity dropped during culture for each sample group and was lower on day 21 in all cultured groups compared to day 0.

In addition, the water content remained unchanged relative to day 0 in the 30% PEG group at all time points (Fig. 3B). In contrast, the water content significantly increased during culture for the other groups.

### Matrix Proteins

The GAG content normalized to the tissue dry weight decreased significantly during culture for all samples cultured in the artificial AF (day 21 compared to day 0, Fig. 3C). Additionally, GAGs were measured in the medium of these samples at all time points (Fig. 3D). At day 3, significantly higher GAG levels were measured in the medium of samples balanced to 0% PEG compared to samples balanced to 30% PEG. Moreover, the GAG content measured in the medium was lower at days 7 and 21 compared to day 3 for all constrained samples.

Furthermore, the free swelling controls contained significantly lower amounts of GAGs in the tissue at days 3, 7, and 21 compared to the 30% PEG group. The medium of the free swelling samples contained the highest amount of GAGs at day 3, which was significantly higher compared to the values measured at day 3 in the 10–30% PEG groups.

Finally, the collagen content significantly increased at day 21 compared to day 0, for all sample groups, including the free swelling samples (Fig. 3E).

### DNA Content

Slight changes in DNA content were observed in the constrained samples during culture (Fig. 3F). The DNA content in the 0 and 10% PEG group significantly decreased between days 3 and 21. Moreover, at day 21, DNA levels measured in the 0% PEG group contained significantly less DNA compared to day 0 controls.

The DNA content also changed in the FS groups during culture. At day 21, the DNA content in the free swelling samples (±310 mOsm) increased to almost 500%, while a decrease of approximately 40% was measured in the 0% PEG constrained group (±310 mOsm). Furthermore, a layer of densely packed cells was observed on the periphery of the free swelling samples (data not shown).

### Inflammatory Markers

**Figure 4:**
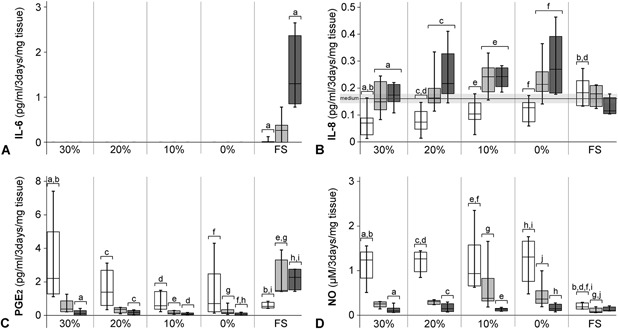
The concentration of interleukin‐6 and ‐8, prostaglandin E2, and nitric oxide in the medium, normalized to the initial tissue weight. Different trends in levels of (A) interleukin (IL)‐6, (B) IL‐8, (C) prostaglandin E2 (PGE_2_), and (D) nitric oxide (NO) were measured in the culture medium of PEG‐treated constrained samples compared to free swelling (FS, unconstrained) controls. X‐axis: 30%, 20%, 10%, and 0% refer to the [PEG] used to pre‐shrink the NP samples, before constraint in the artificial annulus. White bars = day 3, light gray bars = day 7, and gray bars = day 21. See Table S1 for n per group. a,b,c,.. = significantly different (*p* < 0.05) from bars with the same letter.

The inflammatory markers TNF‐α, IL‐1β, IL‐6, IL‐8, PGE_2_, and NO were measured in both the tissue and medium of all cultured samples. However, in the tissue samples, all markers were below the detection limit. In addition, IL‐1β and TNF‐α concentrations were also below the detection limit (31.3 pg/ml and 125.0 pg/ml, respectively) in the medium of all culture conditions at all time points (positive control: IL‐1β stimulated NP tissue). IL‐6 levels were only below the detection limit in the medium of the constrained samples (0–30% PEG groups), while an increasing concentration was measured in the free swelling samples between days 0 and 21 (Fig. 4A).

The concentration of IL‐8 in the medium increased significantly from day 3 to day 21 in all the PEG‐treated groups (Fig. 4B). At day 3, the concentration was below the baseline measurement of the fresh medium for all groups, while at day 21 the concentration increased until above the baseline for the 0% and 10% PEG groups. In the medium of the free swelling samples, the IL‐8 concentration did not change over time and remained in the range of the baseline values of the fresh medium. Furthermore, at day 3 of culture, the IL‐8 concentration in the medium was lower in the 20% and 30% PEG samples compared to the free swelling samples.

The concentration of PGE_2_ in the medium of constrained samples decreased from day 3 to day 21, while it increased for the free swelling samples during this period (Fig. 4C). Finally, the concentration of NO was significantly lower at day 21 compared to day 3 for all constrained samples (0–30% PEG, Fig. 4D). The NO concentration in the medium of the free swelling samples did not change during culture. At day 3, the NO concentration was significantly lower for free swelling samples compared to the constrained samples at this time point. During culture, this difference was reduced, and at day 7 the NO concentration was only significantly lower for the free swelling samples compared to the 0% and 10% PEG samples.

## DISCUSSION

To evaluate if a decrease in tonicity of constrained NP tissue triggers an inflammatory cascade, healthy bovine NP tissues were balanced against different PEG concentrations to obtain a range of tonicities (isotonic to hypotonic). During culture, a short‐term but tonicity‐independent inflammatory response of the NP cells was observed. Surprisingly, only mid‐stream and downstream inflammatory cytokines, for example, IL‐8, PGE_2_, and NO were detected, while the upstream cytokines TNF‐α and IL‐1β, which have been reported as the major inflammatory cytokines of (intact) disc degeneration and herniation, were absent.^6^, ^15^, ^24^ This finding may suggest that osmotic factors directly affect NP cells, and that the inflammation process within the degenerating disc is more complex than the classical inflammatory process induced by loss of tonicity. Alternatively, other upstream cytokines, for example, IL‐17 which can also stimulate the NF‐κβ pathway,^15^, ^33^ may be involved but these were not investigated in this study.

Furthermore, differences in inflammatory reactions were observed between constrained hypotonic (0% PEG) samples and free swelling samples, even though they had similar tonicities. Culture of constrained NP tissues, including the 0% PEG group, resulted in an increase of IL‐8 production and a temporal elevation of PGE_2_ and NO cytokines. IL‐8 has been identified as a potent chemoattractant for neutrophils and lymphocytes and has been associated with the development of radicular pain.^11^ Contrarily, culture of free swelling tissue resulted in a sustained production of inflammatory markers IL‐6 and PGE_2_, similar as reported for herniated tissues.^34^, ^35^ Elevation of IL‐6 is known to trigger several inflammatory pathways that influence numerous processes, inducing growth and differentiation of B‐ and T‐cells.^15^ These observations suggest that NP cells induce different mechanisms as a response to a decrease in tonicity with restricted or free swelling conditions. It may be hypothesized that NP cells stimulate neutrophil and lymphocyte recruitment during disc degeneration with intact AF, while growth and differentiation of immune cells are regulated after herniation. However, the current study focused solely on the NP, while the in vivo inflammatory cascade is likely more complex due to the involvement of body's immune system and the AF. Therefore, we strongly encourage further evaluation of the role and development of inflammation within the two different stages of disc degeneration (with and without intact AF).

In addition, differences at tissue level were observed between constrained hypotonic (0% PEG) samples and free swelling samples. The DNA content of constrained samples decreased to 50% of the initial content, while it increased 600% in the free swelling samples. Indeed, an increase in spindle shaped cells, which formed several cell layers around the tissue periphery, was observed in the free swelling samples, explaining the increase in DNA content. These spindle‐shaped cells were previously identified in vitro as dedifferentiated NP cells^25^, ^36^ and were demonstrated to have a decrease in aggrecan and collagen type II production and an increase in collagen type I production compared to healthy NP cells.^36^ These changes are consistent with observations in NP cells of degenerating discs and herniated canine tissues.^6^, ^35^ The presence of a second cell population in the FS samples may also explain the differences observed in cytokine production between FS and 0% PEG treated samples.

A range of tonicities was obtained at day 3 between the different PEG‐treated NP tissues (320–430 mOsm). In human NP tissue the tonicity reduces during disc degeneration with ∼25% between grade 2 and 3 of the Thompson scale, and ∼35% between grades 2 and 4 (calculated from Antoniou et al., 1996).^37^ The total tonicity difference in our culture system is 25%, indicating that our culture conditions mimic only early stage disc degeneration. In order to mimic tonicities of advanced degenerated NP tissues, the amount of GAGs need to be further reduced via, for example, enzymatic degradation.

Although a tonicity range was obtained at day 3, differences between groups reduced during culture, and thus similar tonicities were observed in all constrained samples at day 21 (310–350 mOsm). Contrarily, differences in the water content due to the PEG procedure remained stable during culture as would be expected by the limited swelling from the artificial annulus. This indicates that the time‐related drop in tonicity, calculated from the amount of proteoglycans per wet weight, is caused by the loss of proteoglycans. Indeed, the GAG content reduced during culture in all sample groups, and relatively high GAG concentrations were measured in the culture medium at day 3. This observation is in contrast to our previous study where the proteoglycan content in the (isotonic) 30% PEG‐treated group could be maintained for 42 days.^26^ In our previous study, a membrane with a smaller pore size was used as a barrier between the NP and the artificial annulus, compared to the membrane used in the current study (100 kDa molecular weight cut‐off compared to 0.2 µm pore size). The lager pores were chosen to allow exchange of (large) growth factors between the medium and the tissue, but likely also allowed small proteoglycans to diffuse from the tissue into the medium.

Furthermore, an increase of PGE_2_ and NO was measured at day 3 when the tonicity range was still present, which decreased again during the remaining culture when the tonicity range was lost. Prolonged elevation of NO has been associated with apoptosis,^38^ which could explain the decrease in DNA content observed in the 0% and 10% PEG groups that both were hypotonic throughout the whole culture period. An alternative explanation could be the interruption of regulatory volume decrease (RVD) during the PEG treatment. In isolated chondrocytes, the process of RVD, activated by sudden hypotonic stress, was shown to take 60 min and resulted in apoptosis and chromatin condensation.^39^ Prior to our culture, the NP samples in the 0% and 10% PEG‐treated groups were subjected to a sudden hypotonic stress for 100 min, to change the tissue's tonicity. Although the chondrocyte‐like NP cells resided in their native, proteoglycan‐rich swelling environment during the PEG treatment, this procedure might have influenced the NP cells. Likely, a combination of both processes contributed to the decrease in cell numbers (DNA content) at day 21 compared to day 3. Although the exact mechanism behind the decrease in cell number is unclear, it is consistent with observations in degenerative discs.^8^

In addition to apoptosis, elevated NO levels are associated with increased matrix breakdown and reduced proteoglycan synthesis.^38^ However, in all constrained groups an increase in collagen content was observed at day 21 compared to the day 0 control. Additionally, the reduction in GAG content can be explained with the leaching of GAGs into the medium, suggesting that the temporarily elevated NO levels at day 3 may not be enough stimulation to alter matrix‐turnover.

## CONCLUSIONS

A short‐term inflammatory response was observed in constrained NPs, with only limited differences in short‐term exposure to a range of isotonic to hypotonic tissue conditions. However, different inflammatory markers were produced by NP cells in constrained and unconstrained tissue with the same hypotonicity. These results suggest that different processes may be activated in NP tissue in degenerating discs (constrained NP) compared to herniated discs (unconstrained NP). Furthermore, upstream inflammatory cytokines identified as key players in disc degeneration (TNF‐α, IL‐1β) were undetectable in all conditions, while mid‐ and downstream cytokines (IL‐6, IL‐8, PGE_2_, NO) were produced by the NP cells, implying that the extracellular environment directly affected NP‐cells or that other upstream mechanisms are involved. This observation challenges the hypothesis that NP cells generate a classical inflammatory cascade within the degenerating disc. Overall, these findings encourage further evaluation of the role of NP cells in the inflammation pathways observed during disc disease. Gaining better understanding of the inflammatory mechanisms will help to identify novel targets for the treatments of disc degeneration.

## AUTHORS' CONTRIBUTIONS

V.M.: writing of manuscript, data interpretation, generated figures. I.A.: study design, experimental work, data interpretation, writing of manuscript. B.vD.: study design, data interpretation, revising manuscript. K.W.K.: study design, critically reviewed manuscript. K.I.: study design, data interpretation, critically reviewed manuscript. All authors have read and approved the final submitted manuscript.

## Supporting information

Additional supporting information may be found online in the Supporting Information section at the end of the article.

**Table S1**. Number of independent samples per group and time point for both the tissue and culture medium.
**Table S2**. Sample groups and wet weight change per group after being balanced against different PEG concentrations.
