# Supplementary material for: Hypotonicity differentially affects inflammatory marker production by nucleus pulposus tissue in simulated disc degeneration versus herniation
Source: J Orthop Res. 2019 Apr 1;37(5):1110–6. doi: 10.1002/jor.24268 (PMC6593810; doi:10.1002/jor.24268)
Supplement: Supplementary file 1 — Table S1. Number of independent samples per group and time point for both the tissue and culture medium. Table S2. Sample groups and wet weight change per group after being balanced against different PEG concentrations. [file JOR-37-1110-s001.docx]

Supporting information

**Hypotonicity differentially affects inflammatory marker production by nucleus pulposus tissue in simulated disc degeneration versus herniation**

Vivian H.M. Mouser, PhD^1^, Irene T.M. Arkesteijn, PhD^1^, Bart G.M. van Dijk, PhD^1^, Karin Wuertz-Kozak, PhD^2, 3, 4^, Keita Ito, MD, ScD^1,5^

***Table S1: Number of independent samples per group and time point for both the tissue and culture medium.***

| **Group** | **Day (tissue sample)** | | | | **Day (medium sample)** | | |
| --- | --- | --- | --- | --- | --- | --- | --- |
|  | **0** | **3** | **7** | **21** | **3** | **7** | **21** |
| 30% PEG | 19 | 4 | 5 | 6 | 15 | 11 | 6 |
| 20% PEG | 19 | 3 | 4 | 5 | 12 | 9 | 5 |
| 10% PEG | 19 | 4 | 4 | 6 | 14 | 10 | 6 |
| 0% PEG | 19 | 3 | 5 | 6 | 14 | 11 | 6 |
| FS | 19 | 3 | 4 | 4 | 11 | 8 | 4 |

PEG = polyethylene glycol, FS = free swelling

***Table S2: Sample groups and wet weight change per group after being balanced against different PEG concentrations.***

| **Group** | **[PEG] at day 0**  **(100 minutes)**  **(w/v)** | **Constraint**  **during culture** | **Wet weight change during PEG procedure**  **(%)** |
| --- | --- | --- | --- |
| 30% PEG | 30% | AA | -40.3 ± 7.1 |
| 20% PEG | 20% | AA | -24.9 ± 7.2 |
| 10% PEG | 10% | AA | 13.1 ± 10.1 |
| 0% PEG | 0% | AA | 71.1 ± 26.0 |
| FS | - | None | - |

Numbers are means ± standard deviations. PEG = polyethylene glycol, AA = artificial annulus^26^, and FS = free swelling
